# High-resolution structure of the M14-type cytosolic carboxypeptidase from *Burkholderia cenocepacia* refined exploiting *PDB_REDO* strategies

**DOI:** 10.1107/S1399004713026801

**Published:** 2014-01-29

**Authors:** Vadim Rimsa, Thomas C. Eadsforth, Robbie P. Joosten, William N. Hunter

**Affiliations:** a Division of Biological Chemistry and Drug Discovery, College of Life Sciences, University of Dundee, Dundee DD1 5EH, Scotland; b Department of Biochemistry, Netherlands Cancer Institute, Plesmanlaan 121, 1066 CX Amsterdam, The Netherlands

**Keywords:** carboxypeptidases, metalloproteins, refinement, specificity, zinc enzymes

## Abstract

A potential cytosolic metallocarboxypeptidase from *Burk­holderia cenocepacia* has been crystallized and a synchrotron-radiation microfocus beamline allowed the acquisition of diffraction data to 1.9 Å resolution. The asymmetric unit comprises a tetramer containing over 1500 amino acids, and the high-throughput automated protocols embedded in *PDB_REDO* were coupled with model–map inspections in refinement. This approach has highlighted the value of such protocols for efficient analyses. The subunit is constructed from two domains. The N-terminal domain has previously only been observed in cytosolic carboxypeptidase (CCP) proteins. The C-terminal domain, which carries the Zn^2+^-containing active site, serves to classify this protein as a member of the M14D subfamily of carboxypeptidases. Although eukaryotic CCPs possess deglutamylase activity and are implicated in processing modified tubulin, the function and substrates of the bacterial family members remain unknown. The *B. cenocepacia* protein did not display deglutamylase activity towards a furylacryloyl glutamate derivative, a potential substrate. Residues previously shown to coordinate the divalent cation and that contribute to peptide-bond cleavage in related enzymes such as bovine carboxypeptidase are conserved. The location of a conserved basic patch in the active site adjacent to the catalytic Zn^2+^, where an acetate ion is identified, suggests recognition of the carboxy-terminus in a similar fashion to other carboxypeptidases. However, there are significant differences that indicate the recognition of substrates with different properties. Of note is the presence of a lysine in the S1′ recognition subsite that suggests specificity towards an acidic substrate.

## Abstract 2

The structure of a bacterial M14-family carboxypeptidase determined exploiting microfocus synchrotron radiation and highly automated refinement protocols reveals its potential to act as a polyglutamylase.

## 1. Introduction   

Carboxypeptidases (CPs) hydrolyse peptide bonds in target proteins to release C-terminal amino acids. These enzymes have been classified into different families according to sequence similarity, mechanism and function (Gomis-Rüth, 2008 ▶; Rawlings *et al.*, 2012 ▶). One of the largest groups, carrying a single catalytic Zn^2+^ in the active site, is the M14 family. This is divided into four subfamilies known as M14A, M14B, M14C and M14D. One of the earliest and best studied family members is bovine carboxypeptidase A (CP-A; Christianson & Lipscomb, 1989 ▶). In general, M14A and M14B carboxy­peptidases either function within the secretory pathway or are themselves secreted. The members of the M14A subfamily are produced as pro-enzymes that are activated following the cleavage of N-terminal segments. The M14B subfamily are produced as active enzymes with a transthyretin-like domain at the C-­terminus. The M14C group consists of bacterial enzymes related to γ-d-glutamyl-(l)-*meso*-diaminopimelate peptidase I (http://merops.sanger.ac.uk/) and process components of the bacterial cell wall. Like their M14A relatives, they also carry an N-terminal extension.

The M14D subfamily has only recently been described and consequently there is only limited information regarding their structure and activity. They are referred to as cytosolic carboxypeptidases (CCPs; http://merops.sanger.ac.uk/) to reflect their cellular location. A search of available genomic data identified CCPs in many organisms (Otero *et al.*, 2012 ▶) and the number of CCP paralogues varies between species (Rodriguez de la Vega *et al.*, 2007 ▶). Four mammalian CCP proteins and a homologue from *Caenorhabditis elegans* called *Ce*CCPP-6 have been identified as deglutamylases (Kimura *et al.*, 2010 ▶; Rogowski *et al.*, 2010 ▶). In addition to this activity, CCPs might process other C-terminal amino acids. For example, one member of the family has recently been reported to cleave the C-terminal tyrosine from α-tubulin (Sahab *et al.*, 2010 ▶). Less is known about the functional activity of the bacterial CCPs. A recent study of *Pseudomonas aeruginosa* CCP (*Pa*CCP) failed to detect any activity towards known CP substrates, and the possibility has been raised that structural changes might be required to generate an active enzyme (Otero *et al.*, 2012 ▶).

Our interest in the bacterial homologues is twofold. It has proven difficult to obtain a recombinant source of mammalian CCP for characterization and therefore the prokaryotic proteins offer a surrogate system to inform on the post-translational modification of tubulin. We also note that in the important Gram-negative pathogen *P. aeruginosa* the gene encoding the CCP homologue (PA2831) has been identified as essential for the establishment of a lung infection in a murine model (Winstanley *et al.*, 2009 ▶), and as such the CCP might represent a potential drug target for an important group of Gram-negative bacteria. As part of a wide-ranging project to advance early-stage drug discovery of novel antibacterials, we have adopted a structure-based approach to drive target assessment (Eadsforth *et al.*, 2012 ▶; Moynie *et al.*, 2013 ▶). Key to this process is to acquire material for ligand-binding studies: an accurate structural model which can be exploited to inform on aspects of druggability and crystals that might assist in fragment screening (Hunter, 2009 ▶). We initiated work on *P. aeruginosa* CCP (*Pa*CCP; UniProt entry Q9I012) and on *Burkholderia cenocepacia* CCP (*Bc*CCP; UniProt entry B4EEQ5). We made no progress with *Pa*CCP, but a study has been published by others (Otero *et al.*, 2012 ▶). Our work with *Bc*CCP was more successful and here we report the preparation of an efficient recombinant protein-production system and protocols for purification and crystallization together with the high-resolution structure. With only small crystals available, it was necessary to exploit a microfocus beamline to obtain the diffraction data. *Bc*CCP consists of 384 amino acids and forms a tetramer. The asymmetric unit comprises four subunits comprising in excess of 1500 amino acids. With a sizable model and high-resolution diffraction data with which to refine it, we sought an efficient route through the refinement process and incorporated the strategies employed in the *PDB_REDO* protocols (Joosten *et al.*, 2011 ▶, 2012 ▶). We compare *Bc*CCP with *Pa*CCP (Otero *et al.*, 2012 ▶), with two other bacterial CCP structures in the Protein Data Bank (PDB) and with mammalian carboxypeptidases of defined substrate specificity. These structures identify the key residues and motifs that are important for the stability of the CCP fold and the architecture of the catalytic centre, reveal the presence of a flexible loop at the periphery of the active site and suggest that the substrate of the bacterial proteins is acidic. Moreover, they support a druggability analysis of the active site.

## 2. Materials and methods   

### 2.1. Reagents, preparation of a recombinant expression system and protein purification   

Chemicals and materials were sourced from Sigma–Aldrich and VWR International, except where stated otherwise. Genomic DNA from *B. cenocepacia* J2315 [Laboratory for Microbiology, Faculty of Sciences, Ghent University (LMG) strain 16656] was the template for PCR with the following primers designed to amplify the *Bc*CCP open reading frame using *Nde*I and *Xho*I restriction sites (bold), respectively: 5′-­**CAT**-**ATG**-ACC-CTT-TCG-ATC-ACC-AG-3′ and 5′-**CTC**-**GAG**-TTA-CGC-GAA-CGT-GTC-3′. The PCR product was ligated into pCR-Blunt II-TOPO (Invitrogen) prior to being subcloned into a modified pET-15b vector (Novagen), which produces a histidine-tagged protein with a *Tobacco etch virus* (TEV) protease site. The hexa-His tag, TEV cleavage site and additional residues from the choice of cloning sites added a total of 24 extra amino acids at the N-­terminus. The recombinant plasmid was amplified in *Escherichia coli* XL-1 Blue cells and the integrity of the gene sequence was verified by DNA Sequencing & Services (Dundee University) before being transformed into *E. coli* ArcticExpress (DE3) competent cells for protein production. Cells were grown in 1 l Luria–Bertani medium supplemented with 50 µg ml^−1^ carbenicillin. Gene expression was induced at 310 K using 1 m*M* isopropyl β-d-1-thiogalactopyranoside and growth continued for 72 h at 286 K.

**Figure 1:**
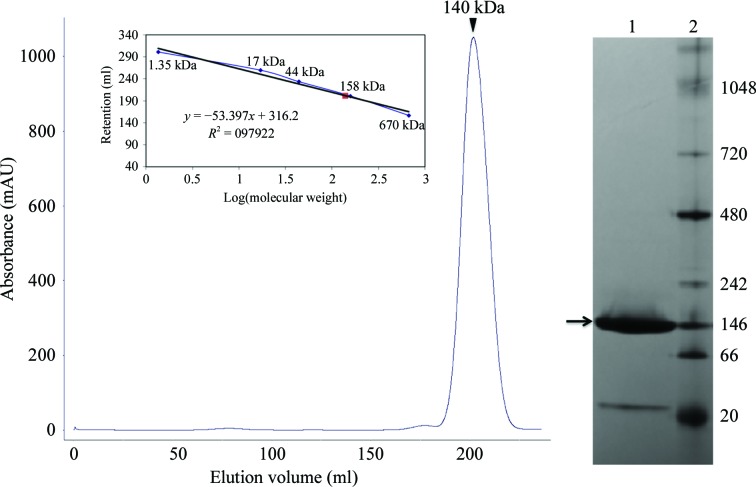
Investigating the quaternary structure of *Bc*CCP. A gel-filtration chromatogram for *Bc*CCP is displayed on the left. The protein eluted with an apparent molecular mass of 140 kDa as calculated from a previously determined calibration curve. Native PAGE analysis of purified *Bc*CCP is shown on the right. The 4–16% native PAGE (Novex) gel shows protein (lane 1) and molecular-mass standards (lane 2; labelled in kDa).

The cells were harvested by centrifugation (30 min at 3500*g* and 277 K), resuspended in lysis buffer (50 m*M* Tris–HCl pH 7.5, 250 m*M* NaCl) containing DNase I (0.1 mg) and a single tablet of a cocktail of EDTA-free protease inhibitors (Roche) and lysed using a French press at 110 MPa. Insoluble debris was separated by centrifugation at 37 500*g* for 30 min at 277 K. The soluble fraction was loaded onto a 5 ml HisTrap HP column (GE Healthcare) and a linear imidazole concentration gradient was applied to elute *Bc*CCP. Fractions were analyzed using SDS–PAGE and those containing *Bc*CCP were pooled. The hexa-His tag could not be cleaved, so protein still carrying the affinity tag was further purified using a Superdex 200 26/60 size-exclusion column (GE Healthcare) equilibrated with 50 m*M* Tris–HCl, 250 m*M* NaCl pH 7.5. This column had previously been calibrated using the molecular-mass standards thyroglobulin (670 kDa), γ-globulin (158 kDa), ovalbumin (44 kDa), myoglobin (17 kDa) and vitamin B_12_ (1.35 kDa) (Bio-Rad; Fig. 1 ▶). Selected fractions were pooled, dialyzed into 100 m*M* sodium acetate, 150 m*M* NaCl pH 5 and concentrated to 10 mg ml^−1^ using Amicon Ultra devices (Millipore). The protein concentration was determined spectrophotometrically using a theoretical extinction coefficient of 73 590 *M* ^−1^ cm^−1^ at 280 nm calculated using *ProtParam* (Gasteiger *et al.*, 2005 ▶). The sample purity was verified by matrix-assisted laser desorption/ionization time-of-flight mass spectrometry and SDS–PAGE (data not shown), with typical protein yields in excess of 20 mg per litre of culture. Native PAGE and size-exclusion chromatography with multi-angle light scattering (SEC–MALS) were used to establish the oligomeric state of the protein. SEC–MALS experiments were performed on a Dionex Ultimate 3000 HPLC system with an inline Wyatt miniDAWN TREOS multi-angle light-scattering detector and an Optilab T-rEX refractive-index detector. SEC–MALS analysis was performed on an Superdex 200 10/300 column (GE Healthcare) equilibrated with 50 m*M* Tris–HCl, 250 m*M* NaCl pH 7.5. Molar masses spanning the elution peaks were calculated with *ASTRA* v.6.0.0.108 (Wyatt).

### 2.2. Crystallization and data collection   

Crystallization trials were carried out with a Phoenix liquid-­handling system (Art Robbins Instruments/Rigaku) and commercially available screens (Hampton Research). 100 nl protein solution (10 mg ml^−1^ in 100 m*M* sodium acetate pH 5.0, 150 m*M* NaCl, 0.5 m*M* ZnSO_4_) was mixed in a 1:1 ratio with reservoir solution and equilibrated against 70 µl reservoir solution at 293 K. Orthorhombic rod-shaped crystals of approximately 30 × 10 × 10 µm in size were observed after 3 d using a reservoir consisting of 0.2 *M* Li_2_SO_4_, 25% PEG 3350, 0.1 *M* bis-tris pH 5.5. A single crystal was transferred into a cryosolution consisting of the original reservoir supplemented with 20% glycerol prior to flash-cooling at 100 K and was then characterized in-house using a MicroMax-007 rotating-anode generator and an R-AXIS IV^++^ dual image-plate detector (Rigaku) prior to storage in liquid nitrogen. X-ray diffraction data extending to 1.9 Å resolution were subsequently collected on the ID23-2 microfocus beamline at the European Synchrotron Radiation Facility (ESRF). Owing to the size of the crystal and its particularly long *c* axis, it was necessary to utilize a microfocus beam of approximately 10 µm and a helical data-collection strategy. Such a data-collection strategy, combined with a short exposure time of 0.6 s and a MAR Research Mosaic 225 charge-coupled device detector, allowed good separation of the reflections, appropriate sensitivity and low X-ray damage, allowing a complete data set to be collected. Integration and scaling of the data were carried out using *MOSFLM* (Leslie, 2006 ▶) and *SCALA* (Evans, 2006 ▶). The space group was *P*2_1_2_1_2_1_, with unit-cell parameters *a* = 62.9, *b* = 85.9, *c* = 289.0 Å. The asymmetric unit consists of four subunits each of mass 45.3 kDa, with approximately 1500 residues in total, a *V*_M_ value of 2.17 Å^3^ Da^−1^ and a solvent content of approximately 45%.

### 2.3. Structure solution and refinement   

The structure was solved by molecular replacement using a polyalanine model of the monomer of a CCP structure from *B. mallei* (*Bm*CCP), which shares 85% sequence identity (PDB entry 3k2k; Joint Center for Structural Genomics, unpublished work). The rotation and translation functions in *Phaser* (McCoy *et al.*, 2007 ▶) revealed the positions of four polypeptide chains with a log-likelihood gain of 6469. Inspection of the molecular packing in *Coot* (Emsley *et al.*, 2010 ▶) showed no steric clashes and suggested that a tetramer, consistent with the SEC–MALS results (discussed below), was present. Rigid-body refinement was carried out in *REFMAC*5 (Murshudov *et al.*, 2011 ▶). The resolution range was set from 144.5 to 2.5 Å and the *R*_free_ value was 33.3% following rigid-body refinement. Ten cycles of restrained refinement were then carried out with the resolution extended to 1.9 Å. The *R*_work_ and *R*_free_ values were 24.6 and 28.6%, respectively. Chain *A* was inspected with electron-density and difference density maps in *Coot* and some missing side chains were added, while those out of density were deleted. Chain *A* was rotated and translated to provide the other three chains in the asymmetric unit and the four Zn^2+^ ions were included. The model was subjected to several cycles of *PDB_REDO* refinement (Joosten *et al.*, 2012 ▶) followed by electron-density and difference density map inspection, rebuilding and inclusion of water molecules and ligands. Translation/libration/screw analysis (TLS; Painter & Merritt, 2006 ▶) was applied to create different group descriptions.

The *PDB_REDO* process consisted of the following steps. (i) The *B* factor was reset to the Wilson *B* followed by ten cycles of TLS refinement using one TLS group per chain. (ii) Seven ten-cycle trial refinements were performed keeping the TLS model established in (i) fixed, using automatic geometric restraint weighting from *REFMAC*5 and trying increasingly loose *B*-factor restraint weights. The optimal weight was 0.30, which is lower than the default. (ii) Six 30-cycle refinements were performed using the fixed TLS model, the optimized *B*-­factor restraint weight and increasingly tight geometric restraint weights. A restraint weight of 0.10 gave the best refinement results. Details of the selection algorithm are presented in Joosten *et al.* (2012 ▶). (iv) Model rebuilding consisting of water deletion, peptide flipping, side-chain rotamer searching and His/Asn/Gln flipping was performed to improve hydrogen bonding (Joosten *et al.*, 2011 ▶). (v) A final refinement consisting of five cycles of TLS refinement starting from the previously refined model, followed by ten cycles of restrained refinement using a *B*-factor weight of 0.30 and trying three different geometric restraint weights (0.05, 0.10 and 0.15) was performed. Refinement with a slightly looser geometric restraint weight than before (0.15) gave the best results.

A further round of map inspections, model fitting and *PDB_REDO* refinement was carried out. The output model was again inspected with the relevant maps and adjusted, further waters were added and multiple conformers were now included at several positions. Further restrained refinement followed, using the *B*-factor and geometric weights established with *PDB_REDO* and a TLS description comprising 12 groups in total. Local noncrystallographic symmetry (NCS) restraints were imposed at the onset of the refinement and were maintained until the final round of calculations. The crystallographic statistics and model geometry are shown in Table 1 ▶. Coordinates and structure factors have been deposited in the PDB with accession code 4b6z. The model geometry was monitored with *MolProbity* (Chen *et al.*, 2010 ▶). Structure superpositions were calculated using *Secondary Structure Matching* (Krissinel & Henrick, 2004 ▶) in *Coot* (using *LSQKAB*; Kabsch, 1976 ▶), and the *Protein Interfaces, Surfaces and Assemblies* (*PISA*) server (Krissinel & Henrick, 2007 ▶) was used to characterize the protein–protein oligomerization interface. Amino-acid sequence alignments were performed with *Clustal Omega* (Sievers *et al.*, 2011 ▶). Active-site volumes were calculated using *ICM PocketFinder* (MolSoft Limited). Secondary structure was assigned using *Coot* and by inspection. Figures were prepared using *PyMOL* (DeLano, 2002 ▶) and *ALINE* (Bond & Schüttelkopf, 2009 ▶).

## 3. Results and discussion   

### 3.1. General comments   

**Figure 2:**
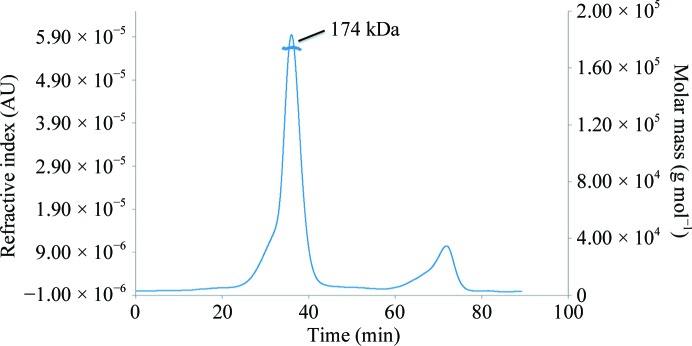
SEC–MALS analysis of *Bc*CCP. The horizontal blue line corresponds to the calculated mass of *Bc*CCP, which was determined to be 174 kDa. The theoretical mass of a monomer is 45.3 kDa; thus SEC–MALS indicates the presence of a tetramer.

**Figure 3:**
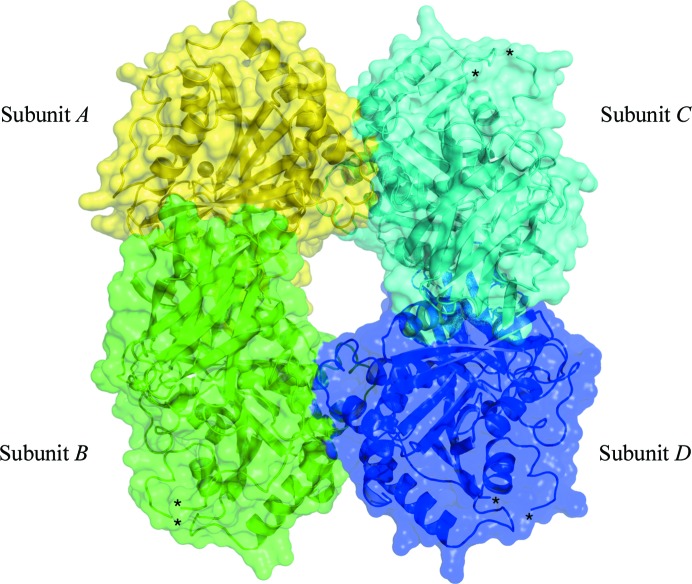
The tetramer of *Bc*CCP. The protein is displayed as a semi-transparent van der Waals surface over the ribbon cartoon. Subunits are labelled *A* (yellow), *B* (green), *C* (cyan) and *D* (blue). Asterisks mark the positions of disordered loops.

Size-exclusion chromatography was used as the final purification step and the protein eluted as a single species with a mass of approximately 140 kDa; a native PAGE gel showed a band of around 146 kDa (Fig. 1 ▶). The theoretical mass of *Bc*CCP is approximately 45.3 kDa; these data were therefore taken to infer the presence of an oligomer, but it was unclear what the quaternary structure was. However, SEC–MALS shows that the protein elutes with a mass of 174 kDa (Fig. 2 ▶) corresponding to a tetramer, and this is consistent with the assembly observed in the crystal structures of *Bc*CCP (the sub­units are labelled *A*–*D*; Figs. 3 ▶ and 4 ▶), *Bm*CCP and the protein from *Shewanella denitrificans* (*Sd*CCP; PDB entry 3l2n; Joint Center for Structural Genomics, unpublished work). This is distinct from *Pa*CCP, which was reported to behave as a monomer in solution (Otero *et al.*, 2012 ▶). The tetramer has a gap or hole at the centre of the assembly and this may have influenced the elution properties, whereas the use of light scattering offers a better approximation of the assembly size.

Diffraction data were acquired by exploiting the use of a microfocus synchrotron-radiation beamline to accommodate the small size of the crystal and to help circumvent the issue of a long unit-cell edge. The structure was solved by molecular replacement and, in order to carry out the efficient refinement of a relatively large asymmetric unit with over 1500 residues and data to high resolution, we adopted a slightly unconventional route by using *PDB_REDO*. This procedure uses a variety of automated protocols to test different models for *B* factors using weighting restraints on geometry, optimization with respect to diffraction data terms and side-chain rebuilding, for example (Joosten *et al.*, 2012 ▶). The approach has been developed to efficiently apply modern refinement techniques to update and improve the contents of the PDB. It has been our experience that the majority of structures that we have inspected have been improved in terms of agreement with the diffraction data and in many cases in chemical correctness. The approach worked well in this refinement and we suggest that the lessons encapsulated in *PDB_REDO* have much to offer especially if incorporated early in the refinement process. We suggest that near the end of the refinement and building process, when interactive rebuilding is limited to the final polishing of the model, it is no longer advantageous to perform subsequent *PDB_REDO* runs. Instead, the optimized refinement settings from the last *PDB_REDO* run can be used in *REFMAC*5 directly. To this end, *PDB_REDO* has been updated to provide a keyword file for *REFMAC*5 that can be used in the *CCP*4 graphical user interface (Winn *et al.*, 2011 ▶) to quickly import these settings.

Continuous well defined electron density is observed for most of the structure. The only disordered regions include the N- and C-terminal residues, residues 157–158 in chains *A* and *C* and a number of residues in a loop (315–320) in subunits *B*, *C* and *D*. Superposition of C^α^ positions of all four chains gives root-mean-squared deviations (r.m.s.d.s) in the range 0.23–0.35 Å, indicating that the subunits are, within the errors associated with the structure, essentially identical. Note that the diffraction-component precision indicator (Cruickshank, 1999 ▶) was calculated to be 0.13 Å. We therefore detail only subunit *A* unless stated otherwise.

A strong feature in electron-density and difference density maps was observed in each active site near Arg226. These were modelled as acetates derived from the 50 m*M* sodium acetate present in the crystallization conditions. In the crystal structure of *Pa*CCP complexed with guanidinoethylmercaptosuccinic acid (Otero *et al.*, 2012 ▶; PDB entry 4a39) and in the structure of CP-A with benzylsuccinic acid (Christianson & Lipscomb, 1986 ▶; Mangani *et al.*, 1992 ▶; PDB code 1cbx) the carboxyl groups of the ligands are located at the same position as this acetate (data not shown).

### 3.2. Subunit structure and comparison with other CCPs   

The structure of *Bc*CCP consists of two domains, a common feature of M14 family members. The primary, secondary and tertiary structures are presented in Fig. 5 ▶. Residues Met1–Glu112 form the N-terminal domain, while the remaining 272 residues make up the CP domain. The N-terminal domain folds into a nine-stranded antiparallel β-sandwich. Strands β2, β3, β5, β8 and β7 form one sheet and strands β1, β4, β9 and β6 form the other. This domain is specific to CCP proteins and is absent in other carboxypeptidases. It has been hypothesized that the N-terminal domain might contribute to folding, might have a regulatory function and/or might be involved in binding other proteins (Kalinina *et al.*, 2007 ▶; Rodriguez de la Vega *et al.*, 2007 ▶). A comparison of the structure of the N-terminal domains of *Bc*CCP and *Pa*CCP gave an r.m.s.d of 0.87 Å over 109 C^α^ positions, indicating close similarity.

The CP domain of *Bc*CCP displays a similar topology of secondary-structure elements, an α/β/α sandwich structure with a seven-stranded antiparallel β-sheet, as observed in other M14 family carboxy­peptidases, for example bovine carboxy­peptidase A (CP-A; Fig. 6 ▶). A superposition of *Bc*CCP and bovine CP-­A (Kim & Lipscomb, 1990 ▶; PDB entry 6cpa), which share about 20% sequence identity, gives an r.m.s.d of 2.03 Å for 225 C^α^ positions. The cores of the structures fit well (Fig. 6 ▶), but on the periphery of the active site there are large differences that will be described later. Overlay of the CP domain of *Bc*CCP with those of *Bm*CCP (PDB code 3k2k), *Sd*CCP (PDB entry 3l2n) and *Pa*CCP (PDB entry 4a39; Otero *et al.*, 2012 ▶) reveals a higher degree of conservation, with r.m.s.d values of 0.45 Å (374 C^α^ atoms), 0.85 Å (362 C^α^ atoms) and 1.07 Å (365 C^α^ atoms), respectively. These proteins share about 85, 50 and 40% sequence identity with *Bc*CCP, respectively, and the degree of structural similarity correlates well with the level of sequence conservation.

*Bc*CCP contains several motifs which are conserved in CCP family proteins. One of them, Asn216-Pro217-Asp218-Gly219, occurs between β13 and α4, with the proline at the start of the helix (Fig. 5 ▶ *b*). This motif contributes to domain folding and the formation of part of the active site (Fig. 7 ▶). On one side of the motif Asp218 establishes hydrogen bonds to the side chains of Thr140 and Arg144 and to the main-chain amide of Glu142. These interactions serve to link β13 to β10 and β11, which are on the surface of the domain. On the other side of the motif Asn216 O^δ1^ accepts a hydrogen bond from the amide of Gly219 to hold the side chain in place and N^δ2^ is a donor in hydrogen-bond formation to the main-chain carbonyl of Gln170 in β12, thus linking different elements of secondary structure together. The side chain of Gln170 also participates in four hydrogen bonds that link β12 to β14 and the segment between α4 and α5. The β-­strand link (not shown) involves the donation of a hydrogen bond from Gln170 N^∊2^ to Asp266 O^δ2^. The other three hydrogen bonds, to the carbonyl of Leu234 and the side chains of Asn235 and Trp238, help to position β12 and the interhelical segment. The positioning of β12 and β14 contributes to the formation of the active site because they provide His171 and His268, respectively, to coordinate the catalytic Zn^2+^.

Mammalian CCP proteins are considerably larger than their bacterial homologues, with N- and C-terminal extensions plus numerous insertions. The sequences of the extended regions vary and their function is at present not known. However, all CCP members share three short sequence motifs within the N-­terminal domain which are absent in other CP families (Kalinina *et al.*, 2007 ▶). These conserved motifs are located in close proximity, even though they are distant in the primary amino-acid sequence (Figs. 5 ▶ *b* and 8 ▶). Motif I, F(D,E)*x*G*x*(L,I), residues 9–14, links β1 with β2 and extends to form part of the second strand. Motif II, W(F,Y)(Y,N)(F,Y), residues 40–43, forms a major part of β4, a buried strand, and with the sequence Trp-Phe-Tyr-Tyr contributes significantly to the formation of the hydrophobic core of the domain together with other aromatic residues such as Phe9, Phe57, Trp69, Trp83, Tyr110 and Phe111 (data not shown). Residues 113–115, P(F,Y)(S,T), form motif III at the N-terminus of α1 on the surface of the domain. This segment forms part of the interface between the N-terminal and CP domains and contributes to interactions with the partner subunits in the dimers (*A*–*B* and *C*–*­D*) forming a tetramer. It is noteworthy that these motifs and features are conserved in the *Pa*CCP, *Bm*CCP and *Sd*CCP structures (Fig. 5 ▶ *b*), and this observation supports the idea that together they are important for the correct folding of the domain and its position with respect to the CP domain.

### 3.3. Quaternary structure   

**Figure 4:**
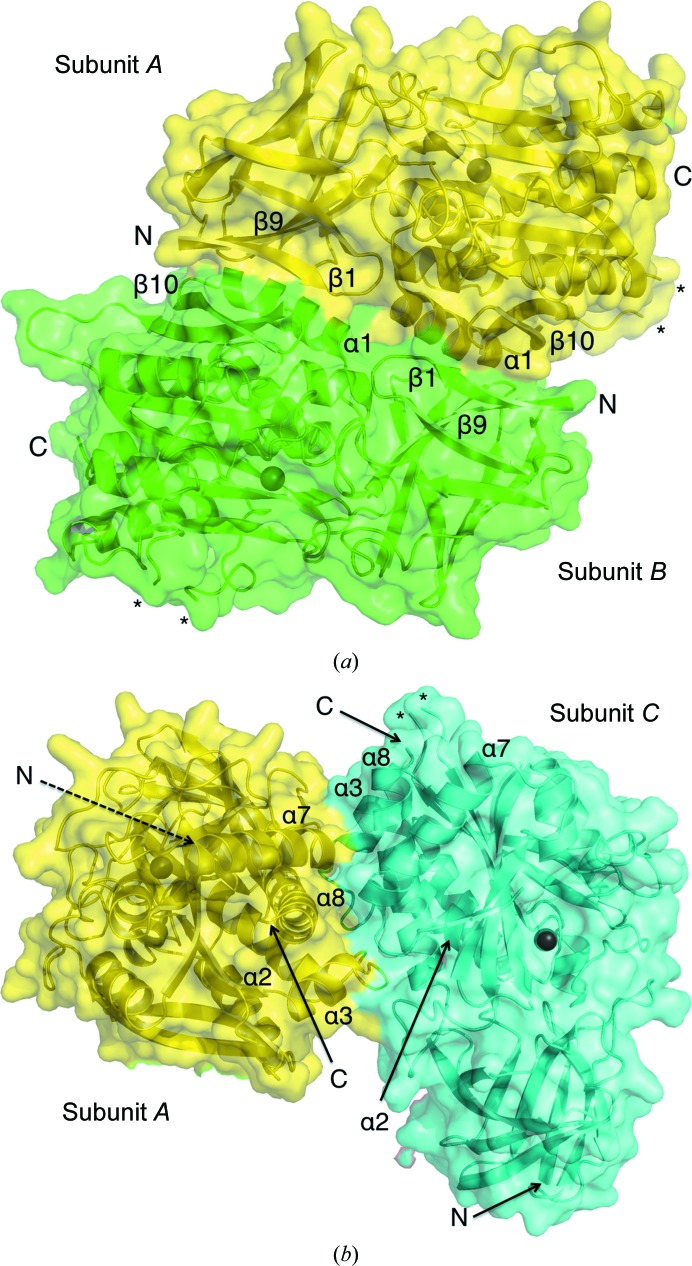
The interfaces between subunits *A* and *B* (*a*) and subunits *A* and *C* (*b*) in the tetramer. The secondary-structure elements which contain residues contributing to tetramer formation are labelled. The N- and C-termini are labelled and the disordered loops are marked with asterisks. The dashed line points to the N-terminal end, which is located at the back and is not visible in the cartoon.

*Bc*CCP forms a tetramer arranged as a dimer of dimers (*A*–*­B* and *C*–*D* in Fig. 3 ▶). The N-­terminus of each *Bc*CCP subunit is located close to the CP domain of the dimer partner (Fig. 4 ▶ *a*). This might explain why the N-terminal hexa-His tag could not be cleaved with TEV protease. The average solvent-accessible surface area of the four subunits in the asymmetric unit is approximately 15 500 Å^2^. The total solvent-accessible area of the *Bc*CCP tetramer is 52 600 Å^2^, with a buried area of 9400 Å^2^. The interface formed between two *Bc*CCP dimers (*A*–*B* and *C*–*D*) occludes 2800 Å^2^, while the interface between subunits *A*–*C* and *B*–*D* equates to 1900 Å^2^. Therefore, about 9% of the surface area of each subunit is involved in dimer formation, with a further 6% involved in associations to assemble a tetramer. The free energy of dissociation is estimated to be 14.9 kcal mol^−1^, suggesting that the tetrameric assembly is thermodynamically stable. These values correlate well with those calculated for tetrameric *Bm*CCP and *Sd*CCP proteins. For example, the total surface areas of the *Bm*CCP and *Sd*CCP structures are 52 500 and 49 900 Å^2^, with buried areas of 11 900 and 9600 Å^2^, respectively. The secondary-structure elements forming the interface within a *Bc*CCP tetramer are shown in Figs. 4 ▶(*a*) and 4 ▶(*b*). Individual *Bc*CCP dimers are stabilized by around 24 hydrogen bonds, nine salt bridges and van der Waals interactions. Some of the residues contributing to dimer formation include Gln127 and Glu117 in α1, Asp104 in a loop between β8 and β9, Ser105 in β9, Arg80 in a loop between β6 and β7, Glu142 in a loop between β10 and β11, Val134 in β10 and Asn8 in β1. We note eight hydrogen bonds and two salt bridges between a pair of dimers in a tetramer. A few of the residues involved in these interactions include Asp193 in a loop between α2 and α3, Lys186 in α2, Arg201 in α3 and Pro354 in a loop between β16 and α8. These residues are not conserved in *Pa*CCP, which is a monomer in solution. However, these residues are well conserved in *Bm*CCP and *Sd*CCP, in which similar subunit–subunit associations are noted in tetramer formation (data not shown).

### 3.4. The active site and specificity   

**Figure 5:**
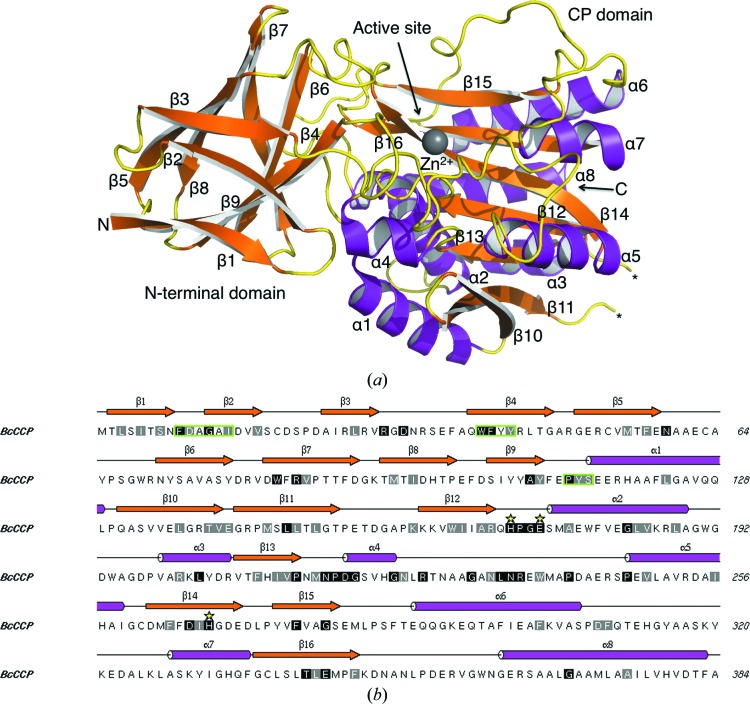
(*a*) The secondary and tertiary structure of *Bc*CCP. A ribbon diagram of subunit *A* showing α-­helices coloured magenta and β-strands coloured orange, with Zn^2+^ depicted as a grey sphere. The N- and C-terminal positions are labelled, as are the α-helices and β-strands. A small section of a loop (residues 157 and 158, marked with black asterisks), as well as the first and last residues at the N- and C-termini, are absent from the model owing to disorder. (*b*) The primary and secondary structure of *Bc*CCP. Residues that are strictly or highly conserved in *Bc*CCP, *Bm*CCP, *Sd*CCP, *Pa*CCP and *Ce*CCPP-6 are shown on black and grey backgrounds, respectively. Three residues that coordinate Zn^2+^ are marked with yellow stars. The three motifs of the N-terminal domain which are conserved in CCP members are enclosed by green boxes.

The active site of *Bc*CCP is an elongated cavity with a base formed by residues in β14, β15 and β16, walls formed by loops linking α4–α5, α6–α7, β3–β4 and β5–β6 and then the β14–β15 turn (Figs. 5 ▶ and 9 ▶). Here, the catalytic Zn^2+^ is coordinated by two histidines (His171 and His268), Glu174 in a chelating η^2^ interaction and a water molecule to give a pentacoordinated distorted tetrahedral geometry (Fig. 10 ▶). The active-site cleft is occupied by an acetate derived from the crystallization conditions. This anion, which marks the binding site for the substrate carboxylate, is held in place by electrostatic interactions with Arg226, Asn235 and Arg236 (Fig. 10 ▶).

The bacterial CCP active sites, and in particular the metal-ion environment (Fig. 11 ▶), display a high degree of structural conservation and this even extends to bovine CP-A, although here the overall sequence identity is only about 20% (see §[Sec sec3.2]3.2). The His171 and Glu174 residues occur in a His-Pro-Gly-Glu motif which is highly conserved in CCP proteins (Fig. 5 ▶ *b*). In *Bc*CCP and *Bm*CCP a buried Arg169 side chain is positioned by a salt bridge to Glu178 and a hydrogen bond to the carbonyl of Tyr114, and in turn helps to stabilize the conformation of the His-Pro-Gly-Glu motif by making a hydrogen bond to the main-chain carbonyl of Pro172 (data not shown). In the other known structures different residues occupy the space near the motif and fulfil the role of stabilizing part of the metal ion-binding site. In bovine CP-A Phe116 provides steric bulk and Gln76 provides the hydrogen bond to the active-site proline to position this metal-binding motif (Christianson & Lipscomb, 1989 ▶).

A proposed mechanism for bovine CP-A involves the activation of a water molecule by the divalent cation assisted by Glu270 acting as a general base and interacting directly with substrate. This supports the generation of a potent nucleophile (Christianson & Lipscomb, 1989 ▶). Arg127 helps to stabilize an oxyanion hole by binding to the substrate carbonyl (Kim & Lipscomb, 1990 ▶). These key residues are conserved as Arg226 and Glu344 in *Bc*CCP (Fig. 11 ▶). Other residues conserved in the active site throughout the M14 family include Asn144 and Arg145 of bovine CP-A, which bind to the C-­terminal carboxylate of the substrate. In *Bc*CCP they are conserved as Asn235 and Arg236.

**Figure 6:**
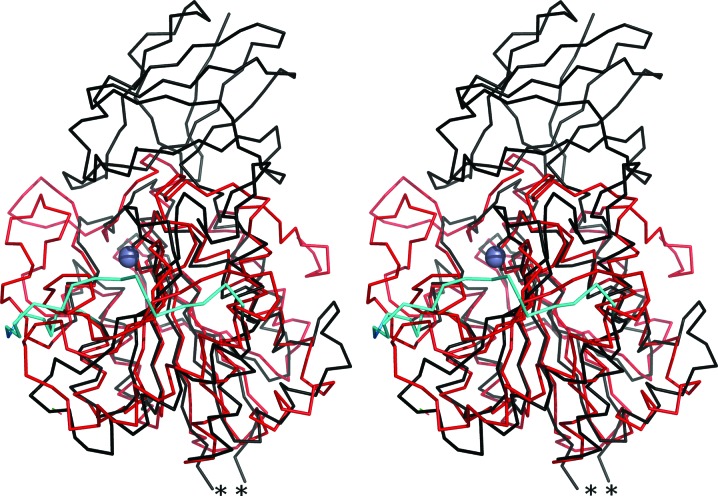
Stereoview C^α^ trace showing the superposition of *Bc*CCP (black) with bovine CP-A (red; PDB entry 6cpa). The α6–α7 loop of *Bc*CCP is drawn in cyan. The active-site Zn^2+^ is shown as a grey sphere and a loop that remains disordered in *Bc*CCP is marked with two asterisks.

**Figure 7:**
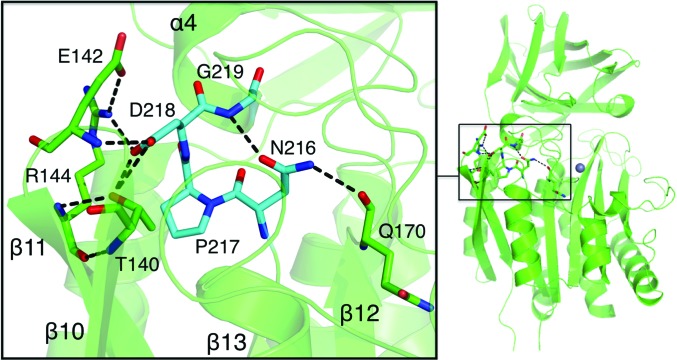
The conserved Asn-Pro-Asp-Gly motif. The left panel shows the residues in stick representation and selected hydrogen-bonding interactions as dashed black lines. The Asn-Pro-Asp-Gly residues are shown in cyan. The right panel shows a ribbon diagram of a subunit to indicate the position of this motif with respect to the overall fold.

Whilst most of the CCP polypeptide chains overlay well, as described, there is a noteworthy exception, part of the flexible α6–α7 loop along one side of the active site, which displays different conformations (Fig. 6 ▶). Similar observations about flexibility and different configurations of this part of the active site have been made when comparing bovine and rat carboxypeptidase structures (Faming *et al.*, 1991 ▶).

The inherent flexibility of this loop is indicated by the thermal parameters. In *Bc*CCP the thermal parameters for the C^α^ atoms in this section, residues His313–Tyr320, average at 27.4 Å^2^ compared with an overall average of 6.7 Å^2^ for all protein atoms. The structural difference is best exemplified by comparing the positions of Lys319 and Tyr320 of the two most conserved structures, *Bc*CCP and *Bm*CCP (Fig. 12 ▶). The *Bc*CCP structure displays an open configuration and the *Bm*CCP structure a closed configuration with Tyr320 directed into the active site. The C^α^ positions of this pair of residues differ by just over 7 Å.

An overlay of *Bm*CCP (PDB entry 3k2k) and CP-A (PDB entry 1cbx), both displaying the closed configuration, places the hydroxyl groups of Tyr320 and Tyr248 within 1 Å of each other (data not shown). In *Bc*CCP Tyr320 is directed out and away from the active site and a major conformational change of about 17 Å would be required to place the hydroxyl in the active site in a similar position to that of Tyr248 in CP-A. In CP-A the tyrosine adopts an outward configuration in the absence of ligand but swings in when a ligand, for example glycyl-tyrosine, is present. Site-directed mutagenesis suggests that it is not a critical determinant of the carboxypeptidase reactivity since *k*_cat_ is unaffected for small substrates. However, a significant increase in *K*_m_ indicates a role in ligand binding, so this tyrosine and the pliable loop in which it resides may assist in the recognition and binding of protein substrates (Gardell *et al.*, 1985 ▶). Subsequent mutagenesis studies led to the proposal that the tyrosine hydroxyl contributes to the mechanism by helping to activate the catalytic Zn^2+^-bound water (Cho *et al.*, 2001 ▶). We cannot find any structural data to support such a conclusion since the functional group on the tyrosine side chain is too distant to make a hydrogen bond to the catalytic water that is bound to Zn^2+^ and there is already a glutamate contributing such a function. Rather, we agree with Gardell *et al.* (1985 ▶) that this tyrosine is well positioned to bind substrates and to assist in fulfilling the hydrogen-bonding capacity of substrates, intermediates or products during the catalytic cycle. There is another tyrosine, Tyr315, positioned in the active site a little closer to the catalytic Zn^2+^ and the associated water molecule. This then provides a tyrosine pair that might contribute to substrate placement in *Bc*CCP.

**Figure 9:**
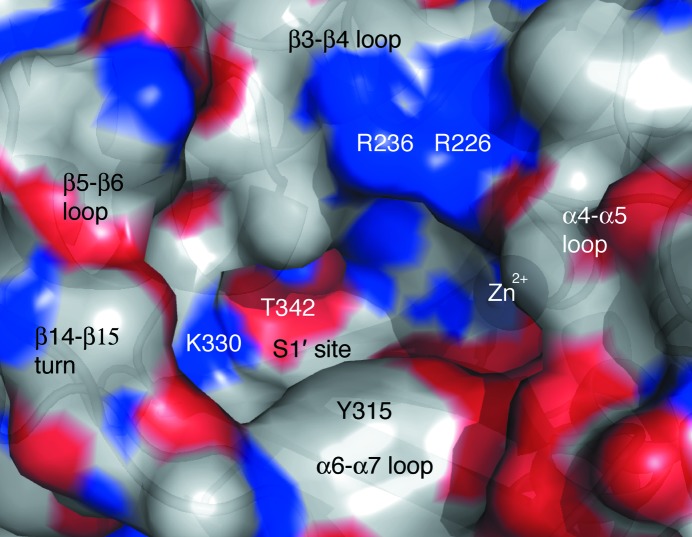
The active-site cavity of *Bc*CCP. The protein is depicted as a semi-transparent van der Waals surface over the ribbon cartoon. The surface is coloured according to atom type: C, white; O, red; N, blue. Zn^2+^ is shown as a grey sphere. Key contributors to the formation of the active site and the S1′ pocket are labelled.

**Figure 10:**
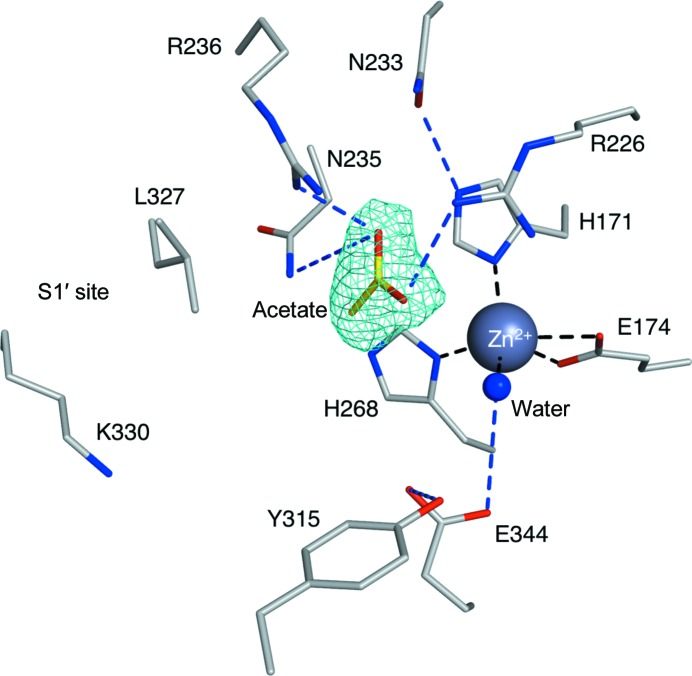
The active site and Zn^2+^ coordination in *Bc*CCP. Selected residues in the active site and acetate are displayed and coloured as follows: C, white (yellow for acetate); O, red; N, blue. The 2*F*_o_ − *F*_c_ electron-density map is shown for acetate, where *F*_o_ represents the observed structure factors and *F*_c_ represents the calculated structure factors. The map is depicted at the 1σ level (cyan chicken wire). The cation and a water ligand are shown as spheres coloured grey and marine, respectively. Zinc coordination is shown by black continuous lines; blue dashed lines represent hydrogen-bonding interactions.

**Figure 11:**
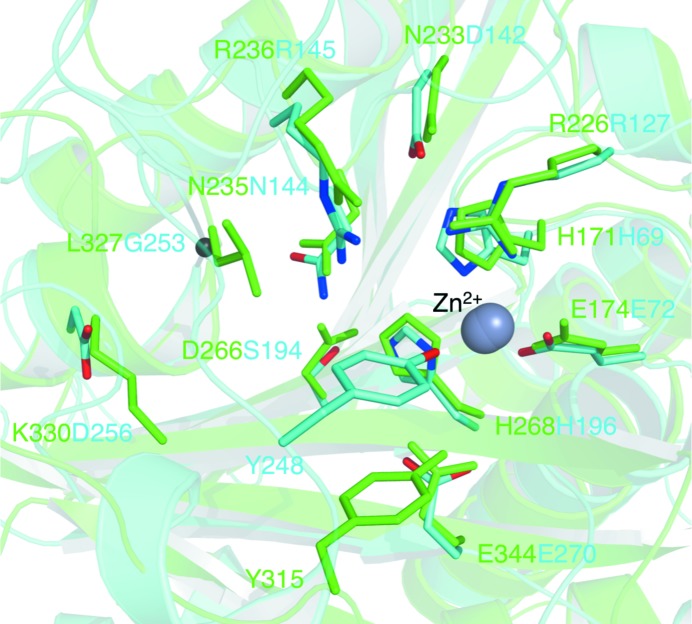
A comparison of the *Bc*CCP and bovine CP-A active sites. The coordinates of CP-A were those of PDB entry 6cpa and the figure is based on the superposition shown in Fig. 2 ▶. Residue side chains are depicted as sticks with atomic positions coloured red for O, blue for N and green for C for bovine CP-A or all in cyan for *Bc*CCP. The C^α^ atom of Gly253 of CP-A is shown as a small black sphere. The catalytic cations are shown as overlapping grey spheres.

**Figure 12:**
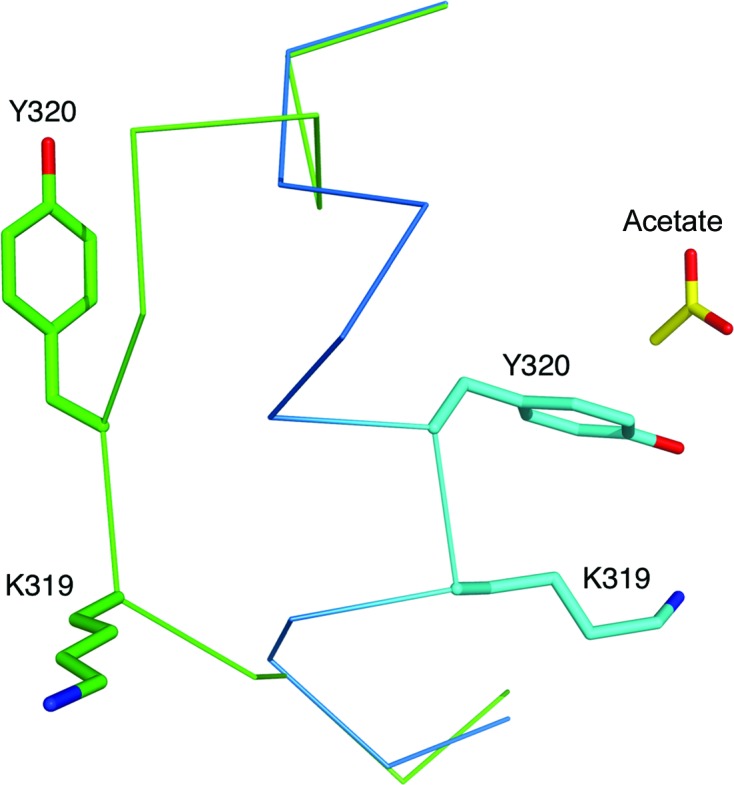
The flexible α6–α7 loop. The *Bc*CCP and *Bm*CCP (PDB entry 3k2k) subunit structures are overlaid and the C^α^ trace for residues 315–326 is shown in green and blue, respectively. The side chains of Lys319 and Tyr320 are shown as sticks.

The substrate specificities of bovine CP-A and rat CP-A2 are well understood (Faming *et al.*, 1991 ▶) and structural differences between CP-A and *Bc*CCP suggest that these enzymes have distinctive specificities. Firstly, it is worth noting that we are comparing a ligand complex of CP-A with unliganded *Bc*CCP. This is relevant in that the α6–α7 loop displays a closed configuration in CP-A, positioned over the active site. The residues in these loops placed in the vicinity of the active site might interact with substrates, but the key determinants of specificity reside in the side-chain binding pocket, the S1′ site itself (Figs. 9 ▶, 10 ▶ and 11 ▶), and are strongly influenced by only a few residues. In CP-A these are Ile243, Asn144, Ala250, Gly253, Ile255, Asp256 and Thr268. Ile243 and Ala250, together with Tyr248, are in the α6–α7 loop capping the S1′ pocket. Asn144 and Thr268 are conserved as Asn235 and Thr342 in *Bc*CCP part-way down to the bottom of the S1′ site. Ile255 in CP-A corresponds to Ser329 in *Bc*CCP and the hydroxyl group makes the bottom of the S1′ subsite polar. Nearby, two differences, Gly253 and Asp256 in CP-A but Leu327 and Lys330 in *Bc*CCP, make the the S1′ subsite much more shallow in the bacterial enzyme and replace an acidic residue with a basic one (Fig. 10 ▶). In bovine CP-A Asp256 directly interacts with a side chain of substrate (Christianson & Lipscomb, 1986 ▶) and by providing an acidic hydrogen-bond accepting capability helps to define the specificity of the enzyme. The side chain of Lys330 in *Bc*CCP extends into the space occupied by Ile243 and Asp256 in CP-A (data not shown). Lys330 of *Bc*CCP is strictly conserved in *Bm*CCP and therefore the S1′ subsite in the *Burkholderia* enzymes is perfectly matched to bind glutamic acid, consistent with the putative function as a polyglutamylase. The lysine is replaced by an asparagine (Asn321) in both *Sd*CCP and *Pa*CCP, so the S1′ subsite would still be cabable of binding glutamate.

To test *Bc*CCP for deglutamylase activity, *in vitro* assays were performed using a furylacryloyl glutamate derivative (data not shown). Only low levels of this substrate were cleaved, indicating that *Bc*CCP might not be a deglutamylase, that the tested ‘substrate’ is not appropriate or that the enzyme might require activation, as is the case for bovine CP-­A.

### 3.5. Issues regarding druggability   

The concept of a druggable protein concerns the properties of a binding site on the target to bind molecules that possess the right physicochemical attributes to make them bioavailable and have a high affinity for the target and a low toxicity to humans (Hunter, 2009 ▶; Fauman *et al.*, 2011 ▶). The optimal target would be a small, well defined and ordered cavity in the protein with pronounced hydrophobic components, although there are notable exceptions. The active sites of enzymes such as pteridine reductase (PDB entry 2x9g; Dawson *et al.*, 2010 ▶) and dihydrofolate reductase (PDB entry 3cl9; Schormann *et al.*, 2008 ▶) would be described as ‘druggable’. The volumes of their active sites are estimated as 350 and 380 Å^3^, respectively. Based on the crystal structures of the proteins discussed here the substrate-binding cavity of bacterial CCP is estimated to occupy a volume of approximately 310–410 Å^3^, which would be judged of a sufficient size to bind drug-like molecules (An *et al.*, 2005 ▶).

Although size matters, the chemical properties of the active site are also critical. In this respect, we note specific functional groups (*e.g* Lys330) and hydrophobic surfaces (tyrosines) that might interact with potential inhibitors. There is, in addition, the presence of the catalytic Zn^2+^ that provides opportunities to incorporate metal-ion affinity into inhibitory molecules.

## 4. Concluding remarks   

The structure of *Bc*CCP has been determined. Only small crystals with a relatively long unit-cell *c* parameter of about 290 Å could be obtained. However, by exploiting the advantages of a microfocus synchrotron beamline we were able to obtain high-resolution diffraction data. An unconventional approach to refinement was adopted by exploiting the strengths inherent in the automated protocols of *PDB_REDO*. This ensured efficient refinement of an asymmetric unit containing over 1500 residues and a large number of water molecules. Our experience was positive and points to a future in which such an approach might become standard. The structure is compared with those of other CCP proteins and with that of CP-A. The CP domain of *Bc*CCP is similar to other M14 family CPs, while the N-terminal domain assembles into a fold unique to CCP subfamily proteins. Our data provide details of the active site and reveal the spatial conformation of residues involved in Zn^2+^ coordination and reaction catalysis. Specific differences localized to the S1′ pocket on one side of the active site suggest that the bacterial CCP proteins process substrates based on the recognition of an acidic residue. This would be commensurate with their assignment as polyglutamylases, although further experiments are required to address this issue.

## Supplementary Material

PDB reference: carboxypeptidase, 4b6z

## Figures and Tables

**Figure 8:**
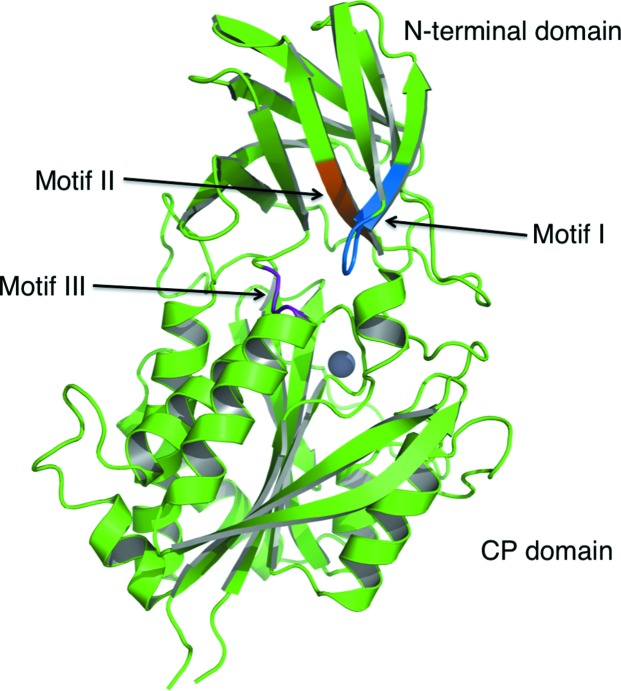
Three motifs common to the CCP family: motif I (marine), motif II (orange) and motif III (purple). The active-site Zn^2+^ is shown as a grey sphere and the three conserved motifs of the N-terminal domain that are found in all M14D family members are shown in marine and are labelled accordingly.

**Table 1:** Crystallographic statistics Values in parentheses are for the highest resolution shell.

Space group	*P*2_1_2_1_2_1_
Unit-cell parameters (Å)	*a* = 62.90, *b* = 85.95, *c* = 289.02
Resolution (Å)	40–1.90 (2.00–1.90)
No. of reflections recorded	516512 (74589)
Unique reflections	124234 (17924)
Completeness (%)	99.9 (100.0)
Multiplicity	4.2 (4.2)
〈*I*/σ(*I*)〉	8.4 (2.7)
Wilson *B* (Å^2^)	13.3
Radiation source and beamline	ID23-2, ESRF
Wavelength (Å)	0.873
Residues	
Subunit *A*	2–156, 159–383
Subunit *B*	2–318, 320–384
Subunit *C*	2–156, 159–314, 320–384
Subunit *D*	1–315, 320–384
No. of waters	1062
No. of glycerol molecules	9
No. of ethylene glycol molecules	7
No. of PEG molecules	1
No. of Cl^−^ ions	3
No. of Zn^2+^ ions	4
No. of acetate ions	4
*R*_merge_ † (%)	13.0 (48.4)
*R*_work_ ‡/*R*_free_ § (%)	16.4/20.5
Mean *B* factors (Å^2^)	
Subunits *A*, *B*, *C*, *D*	6.7
Ligands and waters	26.2
Cruickshank DPI¶ (Å)	0.13
Ramachandran plot††	
Most favoured (%)	96.9
Additional allowed (%)	2.9
Outliers (%)	0.2
Deviations from ideal values‡‡	
R.m.s.d., bond lengths (Å)	0.01
R.m.s.*Z*, bond lengths	0.56
R.m.s.d., bond angles (°)	1.14
R.m.s.*Z*, bond angles	0.62

† *R*_merge_ = 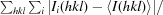 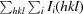, where *I* *_i_*(*hkl*) is the intensity of the *i*th measurement of reflection *hkl* and 〈*I*(*hkl*)〉 is the mean value of *I* *_i_*(*hkl*) for all *i* measurements.

‡ *R*_work_ = 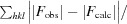 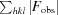, where *F*_obs_ is the observed structure factor and *F*_calc_ is the calculated structure factor.

§ *R*_free_ is the same as *R*_work_ except calculated with a subset (5%) of data that were excluded from refinement calculations.

¶ Diffraction-component precision index (Cruickshank, 1999 ▶).

†† Chen *et al.* (2010 ▶).

‡‡ Engh & Huber (1991 ▶).
